# A Comparison of Phenotypic and Functional Properties of Mesenchymal Stromal Cells and Multipotent Adult Progenitor Cells

**DOI:** 10.3389/fimmu.2019.01952

**Published:** 2019-08-28

**Authors:** Reenam S. Khan, Philip N. Newsome

**Affiliations:** ^1^National Institute for Health Research (NIHR), Birmingham Biomedical Research Centre, University Hospitals Birmingham NHS Foundation Trust, University of Birmingham, Birmingham, United Kingdom; ^2^Centre for Liver Research, Institute of Immunology and Immunotherapy, University of Birmingham, Birmingham, United Kingdom; ^3^Liver Unit, University Hospitals Birmingham NHS Foundation Trust, Birmingham, United Kingdom

**Keywords:** multipotent adult progenitor cell, cellular therapy, mesenchymal stromal cell, immunomodulation, cell biology

## Abstract

Both Multipotent Adult Progenitor Cells and Mesenchymal Stromal Cells are bone-marrow derived, non-haematopoietic adherent cells, that are well-known for having immunomodulatory and pro-angiogenic properties, whilst being relatively non-immunogenic. However, they are phenotypically and functionally distinct cell types, which has implications for their efficacy in different settings. In this review we compare the phenotypic and functional properties of these two cell types, to help in determining which would be the superior cell type for different applications.

## Introduction

Cellular therapy refers to the use of cells to replace or repair damaged tissue/cells. Over the last decade there has been a tremendous development in cellular therapies for the treatment of disease. Embryonic stem cells (ESC) can potentially differentiate into cells of all three germ layers; however, research interest in ESC has been limited by ethical concerns and risk of teratoma formation. Adult cellular therapies have been widely investigated, and haematopoietic stem cell transplantation is already a well-established treatment for various malignant and non-malignant hematological disorders.

Mesenchymal stromal Cells (MSC) and Multipotent Adult Progenitor Cells (MAPC) are both non-haematopoietic cells found in bone marrow stroma, which play a role in maintenance of the haematopoietic stem cell niche (1). Following bone fracture in mice, Park et al. demonstrated mobilization of these cells, and their involvement in fracture repair (2). Great interest in these cells as a potential cellular therapy arises from evidence that they have immunomodulatory properties, can promote angiogenesis, and provide protection against apoptosis. Although MAPC and MSC co-purify, there is evidence that they are phenotypically and functionally distinct cell types.

MSCs were initially described in 1968 by Friedenstein (3), as a subtype of adult fibroblast-like cells with a high proliferative ability, capacity for self-renewal and ability to undergo tri-lineage differentiation to become osteoblasts, chondrocytes and adipocytes. Over time, it became clear that variation in isolation and culture procedures for bone marrow stromal cells contributed to generation of heterogeneous cell populations. The International Society for Cell Therapy (ISCT) subsequently published criteria for identifying MSC: (a) Bone marrow stromal cells that show plastic adherence under standard culture conditions (b) Positive for CD105, CD90, and CD73; have low levels of MHC-I; are negative for MHC-II, CD11b, CD14, CD34, CD45, and CD31 (c) Can differentiate *in vitro* into osteocytes, chondrocytes and adipocytes (4). Thirteen human MSC products have gained marketing authorization, of which nine are for allogeneic therapy and four are for autologous therapy (5), with indications including Crohn's disease, bone and adipose tissue regeneration, graft-vs.-host disease, and acute myocardial infarction.

MAPC were first described several years later, in 2001, as a novel progenitor cell in the bone marrow (6), and whilst these cells meet the ISCT criteria for MSC, they were perceived to be a more biologically primitive population than classical MSC and had greater differentiation potential. Whilst MSCs have been extensively studied, with over 900 clinical trials completed or ongoing, according to the US National Institute of Health (https://www.clinicaltrials.gov), there are fewer data published on MAPC. This review covers a summary of the key similarities and differences in the phenotypic and functional properties of these cells and the clinical data supporting their use in different settings.

## Sourcing the Cells

Whilst MSC were originally identified as a rare population in bone marrow (BM) accounting for 0.01–0.001% of cells (7), they have also been successfully isolated from other tissues including adipose tissue (AT) (8), synovial membrane (9), skeletal muscle tissue (10), dental pulp (11), lung tissue (12), Wharton's jelly (13), umbilical cord (UC) blood (14), amniotic fluid (AF) (15), and placenta (16). Studies have compared the biological properties of MSCs isolated from different sources, and whilst some report that they have similar biological properties (13, 17, 18), others report differences in immunomodulatory activity and surface antigen expression (19–21). Furthermore, UC MSCs have been shown to have a relatively higher proliferative capacity compared to cells from other sources (22), which, has been linked to their having a more primitive phenotype. There is concurrently no consensus on which source of cells is best for clinical application. MAPC were originally isolated from the bone marrow of mice, rats and humans, but subsequently, they were also isolated from murine muscle and brain tissues (6). However, the clinical studies published on MAPC so far have all used cells obtained from human bone marrow.

## Cell Culture and Growth Rates

MAPC and MSC have distinct culture requirements (23). Whilst they are both cultured in fibronectin-coated flasks, MAPC culture medium includes the presence of growth factors (human-platelet derived growth factor, human epidermal growth factor) that are not present in many MSC culture media. Moreover, culture of MAPC takes place in conditions of relative hypoxia (5% oxygen), which is important in preventing telomerase shortening in MAPC. The consequence is that MAPC can be expanded for over 60 doublings without senescence (24), whereas for MSC, the reported population doublings range between 10 and 38 (25). Current manufacturing strategies for MAPC are capable of producing over 100,000 clinical doses from a single donor, sufficient for a clinical trial. Roobrouck et al. (26) demonstrated that the phenotypic and functional properties of the cells were influenced by culture conditions; when MAPC were cultured under MSC conditions, they acquired some of the phenotypical and functional properties of MSC and vice versa (26). Nevertheless, it is important to emphasize that MAPC and MSC are distinct cell types, rather than simply the product of different culture conditions. Following isolation and expansion, both MAPC and MSC can be cryopreserved and stored until needed, although there is evidence that upon thawing, MSCs show signs of injury even within the first 24 h, which may reduce their immunomodulatory properties and increase predisposition to immune clearance (27).

## Cell Phenotype and Issues of Batch-to-Batch Variation

Phenotypically, MAPC and MSC both fulfill the ISCT criteria for identification for MSC (positive expression of CD44, CD13, CD73, CD90, and CD105, negative expression of haematopoietic (CD34, CD45, CD117), and endothelial cell markers (CD34, CD309). They are also negative for MHC class II and co-stimulatory molecules. However, MAPC do not express some of the markers expressed by MSC, such as CD140a and CD140b, for example, and this could be used to distinguish them (26). MAPC also have lower levels of MHC class I and CD44 than MSC and a higher expression of CD49d (28). MAPC and MSC also have distinct features on transcriptomic analysis, with gene signatures that correlate with their specific functional properties (26).

**Figure 1:**
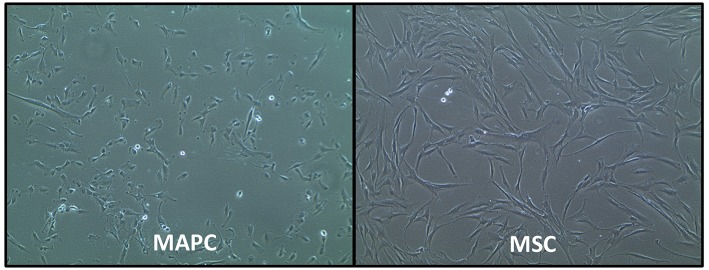
Phase-contrast morphology of human multipotent adult progenitor cells (MAPC) and human mesenchymal stromal cells (MSC). Images courtesy of Regenesys BVBA.

MAPC and MSC also have different morphology, with the former being relatively smaller cells with a trigonal shape, whereas MSC are larger cells with a “spindle”-like morphology [(29); Figure 1]. However, the exact size of MSC does vary according to their source, with placenta-derived MSC being relatively smaller (mean peak diameter 16 μm) than MSC from other sources (30), which are typically >20 μm in size. MSC size is also influenced by their culture conditions. For example, MSC cultured with human platelet lysate (HPL) or platelet rich plasma (PRP) can be smaller than those cultured with fetal calf serum FCS) (30, 31).

Pre-clinical studies of MAPC and MSC have involved a variety of species, including mice, rats and pigs. The genetic profile and cell secretome is slightly different between species, and this has implications for their function. For example, *in vitro*, human MSC (hMSC) proliferation is associated with a very low frequency of oncogenic formation (32), whereas murine MSC (mMSC) frequently gain chromosomal defects (33). The frequency of OCT4 gene expression, which is associated with increased expansion in culture, was found to be relatively higher in rat MAPC (rMAPC) compared with human MAPC (hMAPC) (26).

Clinical trials using purified MAPC have all sourced cells from Athersys. Comparison of MAPC products from different batches has shown minimal batch-to-batch variation by way of surface antigen expression (24), growth rates, effect on suppressing T cell proliferation (34), angiogenic cytokine secretion (25–32% variance for three cytokines between 15 manufacturing runs) (35) and methylation status of 1536 CpG islands (34).

With MSC, there is no single epitope marker that can be reliably used to distinguish them, which results in heterogeneous cell populations across different studies. This makes it difficult to determine whether the effects that are seen are due to an individual cell type from the adherent cell population. Given the heterogeneity of cell types that fulfill MSC criteria, it has been suggested that one method of enhancing the efficacy of these cells would be to sort them on the basis of their expression of specific markers that are associated with favorable characteristics. For example, CD73 positivity on MSC is associated with increased capacity for self-renewal and differentiation (36). The presence of Syndecan-2 (CD-362+) on MSC has been associated with enhanced immunomodulatory properties through downregulation of CD3+ cells by degradation of the T-cell receptor (37).

## Mechanisms of Action

There is evidence that MAPC and MSC can differentiate into cells of mesenchymal lineages, including bones, cartilage, fat, muscles, tendon and bone marrow. Thus, a number of trials have assessed these cells for the treatment of bone and cartilage disease, with several pre-clinical studies showing that MSCs have the potential to stimulate cartilage regeneration and delay joint destruction in osteoarthritis (38). This was supported by findings of a phase I clinical trial, in which patients with osteoarthritis also demonstrated functional improvement (39).

There is also evidence that MSC can trans-differentiate into cells from other lineages, including pancreatic islet cells (capable of producing insulin and glucagon) (40), renal tubular epithelium (41), keratinocytes (42), and hepatocytes although the biological/clinical significance of this is not clear.

MAPC appear to have greater propensity toward endothelial differentiation than MSC (26). In an *in vitro* Matrigel plug assay, human MAPC, but not MSC, could induce functional vessel formation (26). On transcriptomic analysis comparing MAPC and MSC, MAPC had over-representation of genes involved in differentiation of endothelial cells and promotion of angiogenesis, whilst MSC had over representation of genes involved in differentiation of chondrocytes and osteocytes, and in the genes involved in the development and contraction of smooth muscle and genes important for neo-vascularization (26).

However, differentiation is unlikely to explain the predominant functional role of MAPC and MSC. Firstly, experiments used labeled cells have revealed that both MAPC and MSC are rapidly cleared from the body after infusion (43–46), with <1% of MSC retained in the body at 1 week post-infusion. Secondly, upon intravenous injection, the majority of MAPC and MSC tend to get trapped in tissue capillary beds, particularly in the lungs, despite having effects in other organs (47). Thirdly, whilst cell differentiation into non-mesodermal lineages does occur, the frequency of this phenomenon is too low to completely explain the beneficial effects (48). For example, in a pig model of myocardial infarction, Wang et al. injected 50 million MAPC into the heart, and 2 weeks after infusion, only 0.55% of the cells were detectable, and of the engrafted cells, only 2% stained positive for cardiac markers (49). Fourthly, the functional properties of the cells produced through trans-differentiation of MSC and MAPC is questionable. For example, rat MSC that were induced to differentiate into “neural cells” were not able to generate normal action potentials (50), and endothelial cells generated from MSC did not express the same degree of endothelial cell markers as mature endothelial cells (51).

Some studies suggested that the rare reports on cross-germline differentiation of MSC could be ascribed to cell fusion, which may represent an alternative method by which MSC/MAPC can rescue injured cells. MSC can also communicate directly with target cells through delivery of materials, such as mitochondria, via nanotubes or connexins (52). Although there is paucity of mechanistic data on mitochondrial transfer as a putative mechanism of action of MAPC, in a porcine study of intra-cardiac MAPC infusion, treatment was associated with improvement in bio-energetic profiles (46). However, whilst in some studies cell-cell contact has been shown to be important for enhancing activity (53), in a number of studies the beneficial effects of the cell could be, at least partially, reproduced by using components from the cell secretome i.e., the set of factors/molecules released by cells into the extra-cellular space. These include exosomes (30–100 nm), generated from the endocytic pathway and release through exocytosis, whilst microvesicles (50–1,000 nm) are generated through budding from the cell surface and are released from the plasma membrane.

Evidence suggests that MSCs produce large amounts of exosomes in comparison to other cells. These exosomes may be internalized by other cells, permitting release of their contents into the cell cytoplasm (54). Whilst a thorough analysis of the MSC secretome is yet to be performed, it is clear from numerous studies that they express over a 100 proteins, many of which can regulate processes such as immune function, fibrosis, angiogenesis, and apoptosis. Burrows et al. report the only detailed proteomic analysis of the MAPC secretome, in which report identification of 97 proteins. A summary of the key components of each secretome is provided in Table 1.

**Table 1:** Summary of key proteins identified in the secretome of MAPC and MSC that have therapeutic potential.

	**Key components of the MAPC secretome**	**Key components of the MSC secretome**
Chemoattraction/cell adhesion	CXCL1 (55), CXCL3 (55), CXCL5 (35), VEGF (35), sICAM1 (55), SDF1 (56), IL-8 (35)	CCL5 (57), SDF-1 (58), HGF (59), LIF (60), G-CSF (61), VEGF (60), CCL-2 (58), MCP-1 (60), ICAM1 (57), IL-8 (61)
Immunomodulation	IDO (62), TSG-6 (63), PGE_2_ (63, 64), NO (63), semaphorin-7A (55)	IDO (57), PGE_2_ (57), TGF-beta (65), TSG-6 (66), HGF (59), LIF (60), HLA-G (67), IL-6 (61), IL-10 (68), PD-L1 (69)
Neuroprotection	CNF (63), Galectin 1 (55), NO (63)	BDNF (70), NGF (70), GDNF (71), galectin 1 (72), NO
Anti-fibrosis	MMP1 (55), MMP2 (59), TIMP1 (59), TIMP2 (59), cathepsin B (55), bFGF (56)	MMP1 (72), MMP2 (72), MMP7 (72) MMP9, TIMP1 (72), TIMP2 (72), HGF (59), bFGF (73), Ang-1 (58)
Anti-apoptosis	bFGF (56), VEGF (35), versican (55)	VEGF (59, 60), IGF (60), HGF (59), TGFbeta, bFGF (73), GM-CSF (61), IL-6 (61)
Angiogenesis	VEGF (35), CXCL5 (35), IL-8 (35)	VEGF (59, 60), HGF (59), Ang-1 (58), bFGF (73), IGF1 (60), PDGF (73), IL-6 (61)
Anti-bacterial	Pentraxin (55), vimentin (55), lactotransferrin (55)	LL37 (74)
Proliferation	IGFBP4 (55), IGFBP5, IGFBP7, bFGF (56), VEGF (35)	FGF2 (73), VEGF (59, 60), IGFBP3 (60), IGFBP7 (60), IGDBP4 (60), PDGF (73), HGF (59), BMP (72)

*Ang-1, angiopoietin 1; bFGF, basic fibroblast growth factor; BDNF, brain-derived neurotrophic factor; BMP, bone morphogenic protein; CCL2, C-C motif ligand 2; CNF, ciliary neurotrophic factor; CXCL1, C-X-C motif chemokine ligand 1; CXCL3, C-X-C motif chemokine ligand 3; CXCL5, C-X-C motif chemokine ligand 5; CCL5, C-C motif ligand 5; G-CSF, granulocyte- colony stimulating factor; GDNF, glial cell-derived neutrotrophic factor; HGF, hepatocyte growth factor; HLA-G, human leukocyte antigen G; IDO, indoleamine 2,3-dioxygenase; IGF, insulin-like growth factor; ICAM1, intercellular adhesion molecule 1; IGFBP3/4/5/7, insulin-like growth factor binding protein 3/4/5/7; IL-6/8/10, interleukin 6/8/10; LIF, leukemia inhibitory factor; LMCP-1, monocyte-chemotactic protein 1; MMP1/2/7/9, matrix metalloproteinase 1/2/7/9; TIMP 1/2, tissue inhibitor of metalloproteinase 1/2; NGF, nerve growth factor; NO, nitric oxide; PDGF, platelet-derived growth factor; PD-L1, programmed death ligand 1; PGE_2_, prostaglandin E2; SDF1, stromal cell derived factor 1, sICAM1, soluble intercellular adhesion molecule 1; TGF-beta, transforming growth factor beta; TSG-6, tumor necrosis factor inducible gene 6;VEGF, vascular endothelial growth factor*.

Use of conditioned media or extra-cellular vesicles from MSCs had beneficial effects in animal models of myocardial infarction (75), colitis (76), acute liver failure (65), and Parkinson's disease (65). However, clinical experience with exosomes is currently limited. A preliminary clinical study suggested benefit of MSC exosomes in the treatment of stage 4 graft vs. host disease (77), and a clinical trial is underway to test their effect in increasing beta cell mass in patients with type I diabetes (NCT02138331). There have not yet been any clinical trials using cell-free preparations of MAPC, but it would be desirable to further explore cell-free therapy for various reasons. Firstly, it overcomes some of the problems associated with delivery of living cells, including cancer risk, potential for transmission of infections, and immune compatibility. Cell-free preparations would also be easier to store, easier to scale-up, and more cost-effective to prepare. Further, it would be possible to prepare a biological product with a high concentration of the desirable molecules.

**Figure 2:**
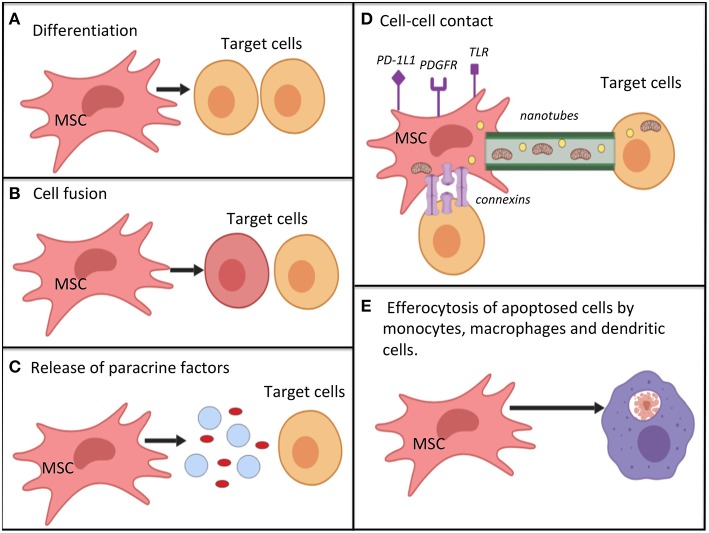
Potential mechanisms by which mesenchymal stromal cells work for immunomodulation, restoration of cell bioenergetics and restoration of cell function; **(A)** differentiation into replacement cell types; **(B)** cell fusion with target cells for rescue of damaged or dying cells; **(C)** secretion of paracrine factors (such as growth factors, cytokines, RNA, and hormones) via micro-vesicles or exosomes. MSC autophagy may help to promote -release of cellular contents; **(D)** cell-cell contact mechanisms. MSC can interact with immune cells via various surface receptors. Transfer of organelles (e.g., mitochondria), ribonucleic acid, and chemicals may occur via nanotubes, or connections; **(E)** efferocytosis of apoptotic MSCs by monocytes, macrophages and dendritic cells. This process causes the phagocytosing cells to adopt a tolerogenic/immunomodulatory phenotype. Mechanisms **(A–E)** are not equivalent, as cell differentiation and cell fusion occur relatively infrequently. MSC, mesenchymal stromal cell; PD-1L1, programmed death ligand 1; PDGFR, platelet derived growth factor receptor; TLR, toll-like receptor.

The secretome of both MAPC and MSC is responsive to changes in their surrounding microenvironment. For example, pre-treatment with IFN-gamma was found to enhance the immunomodulatory activity of MSCs (78), and pre-treatment with TNF-alpha increases their angiogenic effect (79). Therefore, it is possible to pre-condition/prime cells *in vitro* to help in achieving the targetted effects. Pre-treatment of MSC with inflammatory molecules, including IL-1 beta, IL-23, IL-6, and IFN-gamma was found to enhance their immunomodulatory properties in a number of studies (73, 80, 81). Figure 2 summarizes the key mechanisms by which MSC are hypothesized to act.

## Potential for Immune Rejection

In most clinical trials of MSC, and all clinical trials using MAPC, the cells used were allogeneic and were administered without HLA matching or use of immunosuppressive medication. It is commonly believed that these cells are “immune privileged,” leading to the belief that they could be used as a “one-size-fits-all” or “off-the-shelf” therapy. Indeed, cultured MSC and MAPC lack expression of MHC class II and key co-stimulatory surface molecules (CD40, CD80, and CD86), and have low levels of MHC class I molecules, which would help in protecting against a host immune response (82). Additionally, in mixed lymphocyte reactions, both MAPC and MSC do not induce a T-cell response (83).

However, recent evidence has challenged this view as MAPC and MSC are cleared rapidly from the body following infusion. There is evidence that upon exposure to IFN-gamma or upon differentiation, MSC upregulate MHC class I and class II expression (84). MAPC have even lower levels of MHC class I than MSC, which makes them susceptible to lysis by NK cells in their resting state (83). On exposure to IFN-gamma, MAPC upregulate MHC class I but not MHC class II expression, and therefore inflammation may promote MAPC persistence (83).

Evidence from *in vivo* studies also suggests that these cells are not truly immunologically privileged. Eliopoulos et al. administered erythropoietin-transfected MSCs from C57/BL6 mice to syngeneic or allogenic mice (85). The cells were seeded in a collagen scaffold and administered subcutaneously. Whilst the syngeneic mice had a sustained haematocrit response (in response to the erythropoietin), the allogeneic mice had a temporary spike in haematocrit before returning to baseline, suggesting clearance of the MSCs. Furthermore, allogeneic mice, but not syngeneic mice, had CD8+ and NK+ cell infiltration in the scaffolds. In another study, injection of murine luciferase-labeled MSCs to allogeneic hosts was associated with the development of memory T-cells (CD4+, CD122+, CD44+, and CD62L^low^). Tolar et al. used luciferase labeled MAPC to demonstrate that use of T-cell and B-cell deficient mice with NK depletion were all associated with longer persistence of MAPC in mice, suggesting that all three cell types are implicated in rejecting MAPC (83).

In terms of clinical data, allogeneic MSC infusion was found to be associated with development of allo-antibodies in 13% of patients in a phase II clinical trial for GvHD (86). In a phase I clinical trial of allogeneic MAPC in patients with GvHD, infusion was associated with increased serum anti-class I titres compared to baseline, but there was no evidence of MHC class II antibody induction (87).

It has also been hypothesized that the clearance of MSC may be due to triggering of innate immunity independently of HLA-disparity, mediated by a lack of haemocompatibility (88). An “instant blood mediated inflammatory reaction” (IBMIR) has been described previously for pancreatic islet cells and hepatocytes, and is considered to be responsible for loss of up to 80% of these cells shortly after infusion (89, 90). IBMIR is triggered by exposure to host red blood cells and characterized by activation of complement/coagulation cascades, binding of activated platelets to the cells, and clot infiltration by neutrophil granulocytes and monocytes, eventually leading to cell destruction. Moll et al. demonstrated that patients infused with MSC had increased formation of blood activation markers (88). Tissue factor/CD142, which is expressed on MSC, was deemed to be the key determinant of cell haemocompatibility. Tissue factor expression was higher in cells from a higher passage, and for cells administered in higher doses, and there was a donor-to-donor variability. It was also found to vary depending on the cell source. George et al. compared the degree of tissue factor expression for MAPC derived from the bone marrow, bone marrow mononuclear cells, and MSC obtained from different sources (bone marrow, adipose tissue, amniotic fluid, and umbilical cord) (91). They found that tissue factor expression was significantly higher in MSCs originating from the adipose tissue and amniotic fluid, compared with MSC originating from bone marrow and umbilical cord, bone marrow mononuclear cells and MAPC. This would suggest that use of MAPC would be significantly advantageous for intravenous infusion because of the potential for reduced cell clearance/enhanced engraftment.

Thus, MSC are not truly “immunologically privileged,” although rejection occurs slower than it does for other allogeneic cells; thus Aggarwal and Pittenger suggest a better terminology would be “immune evasive” (92). The exact timing and severity of immune rejection is likely dictated by the result of a balance between their immunogenic and immunosuppressive factors, which in turn depends on their local microenvironment. For example, in conditions where there is local immune suppression, for example due to a tumor, the immunogenic properties of the MSC may be masked (93).

Whilst an anti-donor response has been observed, it is reassuring that there have been no adverse events related to immune rejection reported in clinical trials. Furthermore, it is unclear if rejection of allogeneic cells has any impact on efficacy and this is an important area of further work. Indeed, in mixed lymphocyte reactions *in vitro*, bone marrow stromal cells were shown to suppress T-cell proliferation in in a dose-dependent manner, regardless of whether the cells were autologous or allogeneic (53). In a phase II clinical trial of patients with GvHD, there was no difference in efficacy between third-party and HLA-matched MSCs (94), but there are relatively few trials with recorded data on antibody responses. If there is formation of allo-antibodies and T-cell memory in response to allogeneic cells, repeated administration of therapy for a chronic condition may be associated with reduced efficacy.

On the other hand, recent evidence would even suggest that immune response to MSC is crucial to their function. For example, release of complement-activation products following exposure of MSC to host blood can modulate their immunomodulatory and chemotactic activity (95, 96). Contact with activated platelets, as part of the IBMIR response, can also induce extracellular matrix remodeling by MSC, which can potentially contribute to tissue repair (97). Further research is needed to determine the relative importance of IBMIR in the therapeutic efficacy of MSC/MAPC. If the therapeutic benefits on immunomodulation are outweighed by the disadvantages of increased cell clearance/reduced engraftment, it would be helpful to develop strategies to reduce/abolish tissue factor expression on the cells. In fact, given that tissue factor expression is so variable between cells and the potential impact this has on immune clearance, Moll et al. raise the question of whether haemocompatibility should be considered a release criteria for intravascular MSC therapies (98).

The term “autophagy” refers to a system of intracellular degradation that delivers cytoplasmic constituents to the lysosome. This process can be triggered by endoplasmic reticulum stress, hypoxia, and immune cell activation for example. MSC autophagy may serve to either enhance cell survival or promote cell death, depending on the surrounding micro-environment. The induction of autophagy in MSC may promote release of paracrine factors important for their immunomodulatory function (99). Further, recent work by by Galleu et al. and de Witte et al. suggests that following intravenous infusion, MSC accumulate in the lung, where they undergo apoptosis, after which they are engulfed by monocytes, which are subsequently transported elsewhere (100, 101). The process of phagocytosis was demonstrated to induce phenotypic and functional changes in monocytes which resulted in an immunomodulatory response (via release of TGF-beta and IL-10) (101, 102). In contrast, Dang et al. reported MSC autophagy was associated with dampening of their immunomodulatory efficacy (103). These conflicting findings highlight the need for additional studies to investigate this further.

## Potential for Tumorigenesis

There is some evidence that links MSCs to cancer. Long term *in-vitro* culture of murine MSCs was associated with spontaneous transformation of the cells, which were then capable of promoting sarcoma formation when inoculated into immunodeficient mice (33, 104–106). Whilst there were initial reports of a similar phenomenon occurring in human MSCs in prolonged culture, some of these findings were considered to be related it to cross contamination with cancer cell-lines (106, 107). The vast majority of studies report a lack of spontaneous transformation of human MSCs despite extensive culture (108–112). Further, whilst transformation in murine MSCs can be easily induced, particularly through inactivation of *p53* and/or *Rb* genes (113, 114), in human cell lines a number of several, non-physiological, oncogenic events need to be combined for efficient induction of sarcoma (115). Of course, this does not exclude the possibility of cancer formation following infusion in patients, although the risk is low. Rodriguez et al. demonstrated that after hMSC are induced to undergo oncogenic transformation, the transformed cells lose their immunomodulatory and anti-inflammatory properties (116). For example, transformed cells do not secrete the immunomodulatory molecules prostaglandin E2 (PGE2) and PGI2, whereas they do release pro-inflammatory thromboxanes. It is reported that hMAPC remain genetically stable after prolonged culture (34); however, the effects of oncogenic transformation of hMAPC are unknown. In clinical trials, there have been no reports of cancer formation from delivery of allogeneic MSC or MAPC.

Some studies have also researched the interaction between MSC and existing tumors, particularly given that MSC have been demonstrated to home toward tumor sites (117–119). It appears that MSC may promote or inhibit tumor growth, depending on the tumor micro-environment, which as of yet remains undefined (120). The effect of MAPC on cancer growth has not been studied.

## Potential for Thrombosis

Both MSC and, to a lesser degree, MAPC, express tissue factor, with the level of tissue factor expression correlating with pro-coagulant activity (91). The clinical relevance of this for VTE risk is uncertain. Results of functional coagulation assays have been shown to correlate with VTE incidence in multiple patient populations. Thousands of patients have received MSC in clinical trials and there are case reports of thromboembolic events in these patients, but the overall incidence is low (121, 122). Interestingly the case reports of thromboembolism following MSC infusion are related to umbilical-cord derived and adipose tissue derived cells rather than bone marrow derived cells. There have been no published reports of thromboembolic events in clinical trials of patients receiving MAPC infusions, although the available data is much more limited. The true incidence of thromboembolic events with cellular therapies is difficult to establish, partially because several of the patient populations receiving the MSC or MAPC received concomitant anti-coagulation (e.g., low-molecular-weight-heparin, anti-platelet agents and dextran sulfate) for conditions such as myocardial infarction, acute respiratory distress syndrome and stroke. There is also variation in tissue factor activity between cell batches, and between patients.

## Cell Homing and Biodistribution

One of the challenges with bone marrow stromal cell therapy has been targeting their delivery to their intended site of action. Although it is likely that MAPC and MSC exert their effects through paracrine mechanisms, localization to the target site may help in enhancing their efficacy and reducing unwanted peripheral side effects. MSCs are thought to migrate toward inflammatory cues from sites of tissue injury, and in a study of hypoxic ischaemic brain injury in rats, labeled MAPC were detected in the hippocampus regardless of whether they were administered directly into the hippocampus, or intravenously, with similar motor and neurological improvement between the groups (123).

MSC are relatively large cells in comparison to lymphocytes (diameter 15–30 μm vs. 4–12 μm, respectively) (124, 125), which means that they become easily entrapped in smaller blood vessels. The majority of MSC get trapped within the pulmonary capillary bed shortly after intravenous administration, after which they accumulate in the spleen and liver over hours to days (126–130). As MAPC are smaller than MSC, it is unsurprising that, using labeled cells injected intravenously in rats, it was demonstrated that twice as many MAPC were able to pass into the pulmonary circulation compared to MSC (131).

One potential strategy for increasing homing to target organs is intra-arterial rather than intra-venous injection, which has been shown to result in superior bio-distribution outside the lung of both MAPC and MSC (83). However, intra-arterial delivery may result in the cells becoming mechanically trapped in the microvasculature elsewhere (132). Of course, if apoptosis and phagocytosis of MSC in the lung is crucial to their mechanism of immunomodulation, as the work by Galleu et al. and de Witte et al. suggested (100, 101) then reducing pulmonary entrapment may in fact reduce efficacy.

An alternative method to encouraging homing is through cell priming. For example, pre-treatment with TNF-alpha, IFN-gamma, and IL-1 was associated with increased expression of adhesion molecules ICAM and VCAM on MSC (133), and priming with CXCL9 was associated with increased adherence of MSC to endothelial cells (134).

## Role in Immunomodulation

There is extensive evidence supporting the role of MSC and MAPC as modulators of immune responses (Figure 2), with the most well-established effects being on T-cell responses. In mixed lymphocyte reactions, bone marrow stromal cells caused a dose dependent reduction in proliferation of both CD4+ and CD8+ T-cells. When cell-cell contact was prevented, the effects persisted, but were weaker (53). Studies using purified MAPC (53, 135, 136) or MSC confirm that these cells can inhibit T-cell proliferation, with implicated factors including prostaglandin E2 (PGE2), transforming growth factor beta (TGF-beta), inducible nitric oxide synthase (iNOS) and hepHGF (53, 63, 135, 137). Both MAPC and MSC have been associated with changes in the numbers of T-cell subsets, with promotion of expression of T-reg cells (44, 138, 139). Studies also show that MSC may interfere with T-cell function, possibly through secretion of matrix metalloproteinases (MMP), such as MMP-2 and MMP-9 that can cleave CD25 from T-cells (140). The effect of MAPC on B cells has not been widely studied. MSCs have been demonstrated to inhibit B-cell proliferation, alter B cell surface antigen expression and reduce immunoglobulin production (141–143).

MAPC and MSC also affect the innate immune system. MSC have been shown to inhibit NK cell activity, as shown by reduced secretion of IL-15 and IL-2 from the NK cells, with possible mediators including PGE-2 and TGF-beta (144). Macrophages can be crudely classified as being of an M1 (pro-inflammatory) or M2 (anti-inflammatory) phenotype. Both MAPC and MSC have been associated with polarization of macrophages from an M1 (pro-inflammatory) phenotype to an M2 (anti-inflammatory) phenotype (145). This was shown in MAPC an *in vitro* model of axonal dieback (146), and in a murine model of cortical impact injury, in which the authors attributed the increase in M2: M1 ratio to be due to increased apoptosis of M1 macrophages (138). In MSC, this effect is deemed to be due to their secretion of IL-10 and arginase (147, 148). MSC can regulate dendritic cells by interfering with their differentiation to monocytes and inhibiting their activation (149). MAPC can impact immune cell infiltration. MAPC infusions were associated with a reduction of the neutrophil numbers in bronchiolar lavage samples in a sheep model of Acute Respiratory Distress Syndrome (ARDS), and in ischaemia perfusion injury of donor human lungs (150, 151).

Finally, MAPC and MSC can affect the balance of pro-inflammatory and anti-inflammatory cytokines. The secretome of MSC contains both pro-inflammatory (e.g., TNF-alpha, IFN-gamma, and IL-1B) and anti-inflammatory cytokines (e.g., TGF-beta 1, IL-13, and IL-18 binding protein), with the net effect likely to be the result of a balance between the two (43, 151–153). Both MAPC and MSC therapy has been associated with higher levels of a protein called TNF-alpha gene stimulated protein-6 (TSG-6), which can bind CD44 on macrophages and inhibit NfKB activation- the key controller of pro-inflammatory cytokine responses (63).

Clinical studies have investigated the utility of MAPC and MSC in a range of inflammatory and auto-immune conditions. In 2009, the first phase industry-sponsored III trial of MSCs (Prochymal) was completed to investigate their use in treating steroid-refractory GvHD (NCT00366145). The study failed to meet its' primary end-point (complete remission of GvHD 28 days after infusion). However, it was observed that response rates were higher in children, in patients who were treated early, and in patients with gut and liver GvHD. Subsequently a clinical trial was conducted of MSCs in pediatric, severe, GvHD (NCT02336230), although the trial results have not yet been published. Maziarz et al. conducted a phase I dose-escalation study of allogeneic MAPC in 36 patients undergoing myeloablative allogeneic haematopoietic stem cell transplant (87). At day 100, the overall incidences of grade II-IV graft vs. host disease (GvHD) was 37%, but in the group receiving 10 million cells per kg, incidence was 11.1%.

MSC and MAPC have also been investigated in the context of IBD. A phase III clinical trial (NCT01541579) found that allogeneic MSC sourced from adipose tissue were superior to placebo in the treatment of peri-anal fistulas associated with Crohn's disease (154). In a phase II study (NCT01240915) of MAPC in ulcerative colitis refractory to other medical treatments, no significant beneficial effect was seen.

Recent phase II trials suggest that a single MSC infusion in critically ill patients with ARDS is safe, although no impact on mortality was observed (155). Phase IIa trials are in progress to investigate whether MAPC can help in resolution of Acute Respiratory Distress Syndrome (ARDS). A press release from Athersys has reported that preliminary data show a lower mortality and a greater number of ventilator-free days in patients receiving MAPC. Clinical trials of MSC in the context of systemic lupus erythematosus (SLE) (NCT02633163) and diabetes (NCT03484741, NCT02893306, and NCT03343782) are ongoing.

In phase I studies of multiple sclerosis with amyotrophic lateral sclerosis, intrathecal and intramuscular MSC therapy was deemed safe, with possible therapeutic efficacy (156–158). The likely mechanism of benefit is a combination of anti-inflammatory properties of the MSC as well as release of neurotrophic factors.

One of the obvious concerns about using therapies with anti-inflammatory and immunomodulatory properties is the possibility that they increase incidence of infection; however, in clinical trials there has been no reported increased incidence of infection with either MAPC or MSC. To the contrary, there is evidence that MSC and MAPC have anti-microbial effects. In pre-clinical studies, MSC were found to provide protection against sepsis (159–161), whilst MAPC infusions in rats with spinal cord injury were associated with a reduced incidence of urinary tract infection (162). Possible mechanisms of MSC anti-microbial action include release of anti-bacterial peptides (74, 163), and enhancement of the phagocytic activity of neutrophils and macrophages (164). The mechanisms by which MAPC may reduce the incidence of infection are unknown, and this represents an exciting area for future research.

## Role in Angiogenesis

Angiogenesis is the process by which new vasculature sprouts from pre-existing blood vessels. MSC can induce proliferation and migration of endothelial cells promoting tube formation. MSC have been shown to promote angiogenesis in a murine model of cardiac ischaemia reperfusion injury (165). They have also been used to promote angiogenesis in animal models of stroke, myocardial infarction, neurogenic bladder, peripheral artery disease, and stress urinary incontinence (166–168). MSC can secrete both angiogenic and anti-angiogenic factors, and the net result is likely determined by signals from the surrounding environment. For example, exposure to TGF-alpha was shown to increase levels of pro-angiogenic growth factors VEGF, hepatocyte growth factor (PDGF), IL-6 and IL-8 (169). In a murine model of acute limb ischaemic, Ryu et al. found that mice treated with MAPC had higher levels of p-selectin and recruited more Ly6c^lo^ monocytes, which are pro-angiogenic (45). VEGF is an angiogenic factor, which has been identified in the MAPC secretome (55). In a study of MI in pigs, Wang et al. found that conditioned media from the MAPC had higher levels of VEGF, and levels increased further after hypoxia. The authors proposed that this could explain their finding of increased cardiac vascular density in the group treated with MAPC rather than saline (49). Medicetty et al. had similar findings in their pig model of MI (170). In models of critical limb ischaemia in mice, an increase in VEGF, bFGF, and IGF-1 were thought to be responsible for the improved blood flow in mice receiving MAPC injections (171, 172). *In vitro*, serum free conditioned media from MAPC induced endothelial tube formation, but tubes were no longer formed when CXCL5, IL-8, and VEGF were depleted, suggesting the critical role of these proteins (35).

*In vitro* studies suggest that the angiogenic properties of MAPC are superior to MSC. For example, expression of pro-angiogenic proteins GRO, IL-8, and VEGF were found in higher levels in MAPC than MSC, and MAPC had superior functionality in inducing formation of endothelial tubes from human umbilical vein endothelial cells (HUVEC) (26, 173).

In clinical studies, both MAPC and MSC have been investigated in the context of cardiovascular disease. Unsorted bone marrow mononuclear cells have been shown in meta-analysis to be associated with a modest but significant improvement in left ventricular ejection fraction in patients with ischaemic heart disease (174). A recently published meta-analysis including data from 950 patients (across 14 randomized placebo-controlled trials) post-myocardial infarction concluded that MSC therapy was associated with a 3.84% improvement in left ventricular ejection fraction (95% CI 2.32–5.35), and reduction in scar mass by −1.13 (95% CI −1.80 to −0.46) (175). In a phase I clinical trial, Penn et al. found that MAPC treatment in patients following ST-elevation MI was associated with improved ejection fraction (13.5%) and left ventricular stroke volume (25.4 ml), although the study was not statistically powered to detect differences in clinical outcomes (136). Differentiation into cardiomyocytes is a potential mechanism of these effects, but more likely possibilities are that the bone marrow stromal cells promote neovascularization and/or secrete molecules that promote tissue repair (176, 177).

Several clinical trials of bone marrow stromal cells in acute limb ischaemia have been reported, although these have been of limited size and are mainly not placebo-controlled (178). The studies were predominantly of autologous unsorted bone marrow mononuclear cells, rather than purified MAPC or MSC. Overall, there is some data from these (phase I/II) trials that suggests that bone marrow infusions modestly improves ankle-brachial index and pain-free walking distance, but subgroup analysis using data from placebo-controlled trials only shows no significant effect on amputation rate. There is a possibility that the limited efficacy may be related to dysfunctional angiogenesis in the autologous bone marrow cells of patients with established vascular disease (179). Whilst this has been demonstrated *in vitro* for endothelial progenitor cells and bone marrow mononuclear cells, Gremmels et al. showed no difference in angiogenic capacity *in vitro* in MSCs from patients with critical limb ischaemia vs. healthy participants (180). Further work would be needed in the way of large randomized, placebo-controlled trials to determine which type of cellular therapy (MAPC vs. MSC vs. unsorted bone marrow mononuclear cells) would be most efficacious and whether allogeneic therapy has any therapeutic advantage over autologous.

## Role in Fibrosis

Fibrosis is associated with organ failure and high mortality. It is characterized by aberrant accumulation of myofibroblasts, which secrete extracellular matrix proteins like collgen and fibronectin. MSCs have been investigated for their role in reducing fibrosis in the kidney, lung, heart, skin, liver, and bone marrow.

MSCs have been shown to reduce fibrosis in a model of bleomycin induced lung fibrosis (143), and this effect could be reproduced using conditioned media from MSC (181). Cahill et al. showed that MSC promoted fibroblast migration to areas of lung injury, but also inhibited fibroblast proliferation and activation (182). Possible mechanisms include secretion of hepatocyte-growth factor by MSCs, increased levels of MMP expression, and inhibition of TGF-beta. In pre-clinical models, MSC have also been demonstrated to improve dermal fibrosis, with a reduction in alpha-sma-positive myofibroblasts and downregulation of TGF-beta, type I collagen and heat-shock protein 47 expression (183). Pre-clinical models also provide evidence of MSCs having anti-fibrotic effects in the liver, with associated reductions in TGF-beta and alpha-sma expression (184). In early phase clinical trials, there is demonstrable benefit of MSCs on liver biochemistry and MELD score, although evidence of histological benefits is lacking (185). Of course, MSCs have the potential to differentiate into fibroblasts, and so there have been concerned raised about their potential to worsen liver fibrosis; however, there is no evidence of worsening of liver fibrosis on adoptive transfer in clinical trials (185).

In a phase I clinical trial of patients with MI, MAPC were associated with reduced myocardial scarring (136), however, there is little else published clinical data of the effects of MAPC on fibrosis. *In vitro* data show that MAPC do not secrete hepatocyte-growth factor, which does play a role in the anti-fibrotic effects of MSC. However, the MAPC secretome contains a number of factors that could potentially help in reversing fibrosis. For example, MAPC secrete a number of inhibitors of TGF-beta, such as Follistatin-related proteins 1 and 3, and vasorin (55).

## Cytoprotective/Anti-Apoptotic Effects

Both MAPC and MSC have been shown to have anti-apoptotic effects. MSCs can protect against apoptosis by decreasing pro-apoptotic factors like Bax and cleaved caspase 3 expression, whilst increasing anti-apoptotic factors such as Bcl-2 (186).

*In vitro*, MSC and their exosomes have been shown to have high resistance to oxidative stress due to their constitutive expression of a number of anti-oxidant enzymes such as catalase (187). Consistent with this, MSC have been shown to protect hippocampal neurons against oxidative stress caused by amyloid oligomers in a rat model of Alzheimer's disease, which was considered to be due to release of catalase from extra-cellular vesicles, as well as secretion of IL-10, IL-6, and VEGF (188).

Traumatic brain injury usually reduces spleen size, yet rats with traumatic brain injury receiving, MAPC had preservation of their spleen size, which was associated with increased splenocyte proliferation and reduced splenocyte apoptosis (as shown by reduction in caspase 7 and caspase 12 levels on PCR) (153). Pigs receiving MAPC following induced MI had reduced cardiomyocyte apoptosis (46, 49), which was associated with reduced cytochrome C release from cells and downregulation of mitochondrial oxidative enzymes, suggestive of protection of oxidative stress. In addition there was differential expression of genes relating to metabolism and apoptosis detected on gene array (46, 49). When oligodendrocytes were exposed to sub-lethal volumes of hydrogen peroxide, subsequent co-culture with MAPC helped to prolong oligodendrocyte survival, again suggesting that they can protect against oxidative stress (63).

Conditioned media from MSC and MAPC contains numerous neurotrophic factors (189, 190). However, MAPC and MSC have distinct molecular mechanisms for neuroprotection. For example, in pre-clinical studies, it has been shown that tissue inhibitor of metalloproteinase 3 (TIMP3), released by MSCs, plays a critical role in protection against traumatic brain injury by enhancing neuronal survival and neurite outgrowth (191, 192). However, in a rat model of spinal cord injury, in which MAPC were administered with or without TIMP3, the presence of TIMP3 was actually associated with abrogation of MAPC's beneficial effects on tissue sparing and functional recovery (162). It was hypothesized that this may be because TIMP3 may interfere with MAPC migration to the site of injury (162).

In phase I clinical studies, MSC infusion was associated with a variable degree of functional improvement after spinal cord injury, associated with increased serum levels of brain-derived neurotrophic factor, glial-derived neurotrophic factor, ciliary neutrophic factor and neurotrophin 3 and 4 (193–196).

In a phase II double-blinded randomized controlled clinical trial of MAPC (Multistem) in stroke (MASTERS trial), patients with anterior circulation infarct received either Multistem (*n* = 67) or placebo (*n* = 65) (152). Whilst there was no difference in global stroke recovery at day 90, patients receiving the infusions earlier (at <36 h post-stroke) had greater improvement that those receiving the therapy at 36–48 h, and further trials are planned.

## Current Challenges/Future Directions

Bone marrow stromal therapies are a very exciting field for research at present, with evidence showing a range of pleiotropic effects on immunomodulation, fibrosis, apoptosis, and angiogenesis. A summary of the key similarities and differences between MAPC and MSC are shown in Table 2.

**Table 2:** Summary of comparison of key characteristics between multipotent adult progenitor cells and mesenchymal stromal cells.

	**MAPC**	**MSC**
Main sources of cells used in clinical studies	Bone marrow	Bone marrow, adipose tissue, umbilical cord and placenta
Size	<16 μM	>16 μM
Morphology	Smaller, triangle-shaped	Larger, spindle-shaped
Surface markers	CD44^low^, CD45–, CD49d+, MHC1^low^	CD44+, CD45–, CD73+, CD90+ CD105+, MHC1+, CD140+
Culture conditions	Hypoxia, with platelet-derived growth factor and epidermal growth factor	Normoxia, usually without platelet derived growth factor and epidermal growth factor
Immunogenicity	Low	Low
Limit of population doublings (whilst maintaining telomere length and cytogenetic stability)	~60	~10–38
Number of donors required for clinical dosing in trials	Single	Multiple
Haemocompatibility	Relatively high (associated with low tissue factor expression)	Relatively low, particularly for adipose-tissue derived and umbilical cord derived cells (associated with high tissue factor expression)
Potential for immunomodulation	Yes	Yes
Potential for angiogenesis	Yes (likely more than MSC)	Yes
Potential for anti-fibrotic effects	Very limited testing	Yes
Potential for anti-apoptotic effects	Yes	Yes
Safety	Yes (phase I and II clinical trials)	Yes (phase I, II, and III clinical trials)

*MSC, mesenchymal stromal cells; MAPC, multipotent adult progenitor cells*.

Evidence so far from hundreds of clinical trials suggests that MAPC and MSC both have a favorable safety profile. Nevertheless, the concern remains that because the cells' activity is so dependent on surrounding stimuli, there is a possibility that they will have unpredictable side effects *in vivo*.

MSCs have been much more widely studied than MAPC. However, given the heterogeneity in cell types labeled as MSC, comparing study results is difficult. There are very few studies in which properties of MAPC and MSC have been directly compared in the same hands, using the same lab materials, such as culture media. Indeed, it may be possible that some of the trials using early-culture MSC, were in fact MAPC.

Whilst it is become clearer that MSC and MAPC are not truly immunologically privileged, their immune evasive nature gives these therapies particular advantage in acute conditions in which it may not be possible to predict the timing of the insult, and delays in delivery of cellular therapy could retract from its potential benefit. However, for use in cases such as bone transplantation, immune compatibility is more critical. A clear comparison of the efficacy of autologous vs. alloogeneic therapy in various clinical conditions is necessary. It would also be imperative to determine whether the negative impacts of IBMIR reaction can be overcome through use of low-passage cells, and what influence this has on the immunomodulatory activity of the remaining cells.

Whilst there is extensive *in vitro* and pre-clinical data supporting the efficacy of MSC and MAPC, the progress of therapy through clinical trials has been slow. There are relatively few clinical studies of MAPC, whilst hundreds of clinical trials are being performed for MSC with many of them have promising findings, the majority of these are phase I and II trials.

So far, data would suggest that the immunomodulatory and cytoprotective capacity of MAPC is equivalent to that of MSC, and that MAPC may have superior angiogenic and broader differentiation properties. In practical terms, MAPC offer the distinct advantage over classical MSC that they can be produced on a large scale, in a reproducible manner.

The use of cell-free preparations would be preferable to the administration of whole cells, and data with MSC suggests that this could be done without significant loss of efficacy. However, such data are not yet available for MAPC and in both cases, there is a need for good manufacturing practice guidelines for the large-scale production of MSC and MAPC derived products, such as exosomes.

The optimal dosing of both cellular therapies is unknown, and in clinical studies a large variation in dosing has been used. In a phase II clinical trial of patients with stroke, up to 1.2 billion cells were administered per patient (152), with no dose-related adverse effects. However, it would be important, for both cost-effectiveness of therapy and safety, to establish the minimum effective dose, which cannot be extrapolated from animal data.

## Author Contributions

PN had the original concept, provided intellectual input and edited the manuscript, and guarantor. RK wrote the first draft of the manuscript. All authors reviewed the final version.

### Conflict of Interest Statement

PN reports consultancy/speaker fees on behalf of the University of Birmingham from Boehringer Ingelheim, Dignity Sciences, Intercept, Johnson and Johnson, Novo Nordisk, and Shire. His institution receives grant funding from Pharmaxis and Boehringer Ingelheim. RK was supported by a grant from the UK Medical Research Council.
